# Diagnostic and prognostic value of procalcitonin for early intracranial infection after craniotomy

**DOI:** 10.1590/1414-431X20176021

**Published:** 2017-04-20

**Authors:** Y. Yu, H.J. Li

**Affiliations:** 1 Department of Infection, Municipal Hospital of Taizhou, Jiaojiang, Zhejiang, China; 2 Department of Neurology, Municipal Hospital of Taizhou, Jiaojiang, Zhejiang, China

**Keywords:** Intracranial infection, Procalcitonin, Cerebrospinal fluid procalcitonin, Craniotomy, Diagnosis

## Abstract

Intracranial infection is a common clinical complication after craniotomy. We aimed to explore the diagnostic and prognostic value of dynamic changing procalcitonin (PCT) in early intracranial infection after craniotomy. A prospective study was performed on 93 patients suspected of intracranial infection after craniotomy. Routine peripheral venous blood was collected on the day of admission, and C reactive protein (CRP) and PCT levels were measured. Cerebrospinal fluid (CSF) was collected for routine biochemical, PCT and culture assessment. Serum and CSF analysis continued on days 1, 2, 3, 5, 7, 9, and 11. The patients were divided into intracranial infection group and non-intracranial infection group; intracranial infection group was further divided into infection controlled group and infection uncontrolled group. Thirty-five patients were confirmed with intracranial infection after craniotomy according to the diagnostic criteria. The serum and cerebrospinal fluid PCT levels in the infected group were significantly higher than the non-infected group on day 1 (P<0.05, P<0.01). The area under curve of receiver operating characteristics was 0.803 for CSF PCT in diagnosing intracranial infection. The diagnostic sensitivity and specificity of CSF PCT was superior to other indicators. The serum and CSF PCT levels have potential value in the early diagnosis of intracranial infection after craniotomy. Since CSF PCT levels have higher sensitivity and specificity, dynamic changes in this parameter could be used for early detection of intracranial infection after craniotomy, combined with other biochemical indicators.

## Introduction

Intracranial infection is one of the common clinical complications after craniotomy, and causes insurmountable obstacles in neurosurgery (1,2). Intracranial infection directly affects the prognosis of patients with high mortality and morbidity rate (3–5). Earlier reports indicate a 2.6 and 0.5% prevalence of intracranial infection after craniotomy in China (6), and USA, respectively (7). Timely diagnosis of intracranial infection after craniotomy is the key to improve the prognosis of patients. In the premise of effective anti-infection treatment, how to accurately identify the infection is particularly important. Traditional indicators, such as white blood cells and neutrophil count provide an important reference point for infection and clinical judgment. However, their specificity might not be high. Studies have shown that blood leukocyte and neutrophil counts after acute stroke are similar in patients with and without infection (8,9).

The smear and bacterial culture of cerebrospinal fluid (CSF) are considered the gold standard for the diagnosis of intracranial infection, and identification of the type of infection depends also on clinical evaluation. Routine biochemical and bacteriological examination of CSF includes: CSF cell count, levels of protein, glucose and chloride ion, analysis of CSF smear, bacterial culture, and polymerase chain reaction (PCR) (10). These tests have high sensitivity but low specificity, so it is difficult to identify the type of intracranial infection efficiently.

Procalcitonin (PCT) is the propeptide of calcitonin, a glycoprotein without hormone activity. PCT is commonly considered an endogenous nonsteroidal substance (11,12). Procalcitonin is very low under normal conditions (around 0.0025 g/L), and cannot be detected by the conventional test method (13). However, the occurrence of pathogen infection stimulates the parathyroid cells to synthesis and secrete large amounts of PCT into the blood, and this leads to an increase in serum PCT (14). Studies have demonstrated that PCT could be used as a relatively specific indicator in the diagnosis of bacterial infection, especially in pneumonia (15,16). A research study indicated that the sensitivity and specificity of PCT were high and it could be widely used as an early inflammatory marker in clinical diagnosis (17). Their study also indicated that PCT is superior to the traditional markers of inflammation in early diagnosis of bacterial infection and could estimate the infection’s severity and prognosis.

The purpose of this study was to evaluate the early diagnostic potential and prognostic value of PCT and the dynamic changes in PCT in patients with intracranial infection after craniotomy.

## Patients and Methods

### Patients

Patients who underwent craniotomy and were admitted to the intensive care unit (ICU) of our hospital from December 2013 to June 2016 were recruited for this study using the following criteria: 1) patients with clinical manifestations of intracranial infection such as continuous temperature more than 38.5°C, or the temperature normalization after treatment and later increase to more than 38.5°C, postoperative severe headache, vomiting, aggravated unconsciousness, and the occurrence of neck stiffness; 2) patients with potential risk factors of intracranial infection, such as catheter drainage and drainage of ventricle aneurysm cavity, long surgical time, artificial material implantation, and cerebrospinal fluid leakage after surgery; and 3) the exclusion of other infections.

The cerebrospinal fluid of patients was sampled for microbial culture multiple times and antibiotic treatment was administered for infection control at the same time.

This study was approved and registered at the Ethics Committee of Municipal Hospital of Taizhou in October 2015. All subjects gave written informed consent for sample collection. All procedures were undertaken following the requirements of the Declaration of Helsinki.

### Standards for intracranial infection diagnosis

The diagnosis of intracranial infection defined according to the standards issued by the National Ministry of Health is as follows: 1) presence of clinical manifestation of intracranial infection; 2) presence of risk factors, such as HIV/AIDS, hematopoietic stem cell transplant, lymphoid malignancies, neutropenia, hereditary immune defects, etc, and patients with drainage or cerebrospinal fluid leakage (18); 3) inflammatory indicators in cerebrospinal fluid: white blood cell count (WBC) >10×10^6^/L; glucose levels <2.25 mmol/L; chloride <120 mmol/L, and protein >0.45 g/L; 4) positive results for bacteria in cerebrospinal fluid culture. Patients with criteria 4 can be diagnosed individually. Patients with negative CSF culture result but positive for the first 3 diagnostic criteria are also diagnosed as having intracranial infection.

Patients were not included if they had other infections, or with recurrent intracranial infection in the ICU after craniotomy.

### Sample collection and tested indicators

Body temperature was recorded, and CSF and peripheral venous blood samples were collected on the day the infection was suspected and on the 1st, 2nd, 3rd, 5th, 7th, 9th, and 11th subsequent days. The highest temperature reading in each day was noted. CSF samples were obtained by the following ways: 1) lumbar puncture; 2) lumbar cistern drainage; 3) ventricular drainage, and 4) ventriculoscopy. All samples were collected under aseptic conditions and no CSF replacement operation was performed.

The collected CSF sample (6 mL) was divided into 3 equal parts for the following examinations: WBC, measured by modified bovine abalone counting board with the manual microscopic counting method; cerebrospinal fluid protein content, determined by the end-point method; glucose content, by the glucose oxidase method; chloride content, by the selective electrode method; PCT content, by the immunofluorescence double antibody sandwich method (used also for blood samples). In blood samples, C reactive protein (CRP) was detected by immune turbidimetry methods, WBC and neutrophil count by flow cytometry (percentage of neutrophils). The acute physiology and chronic evaluation (APACHE II) score and sequential organ failure assessment (SOFA) scores were recorded daily.

### Grouping

The recruited patients were divided into infected group and non-infected group. Generally, it takes 7 to 10 days to make sure the infection is completely under control. Based on the results of the intracranial infection treatment, patients were divided into infection controlled group (improvement of clinical symptoms and of laboratory tests) and infection uncontrolled group (laboratory parameters did not improve, and CSF culture persisted positive).

### Statistical analysis

SPSS 20.0 software was used for statistical analysis (USA). Normally distributed data were compared using the *t*-test, and the median values of non-normally distributed measurement data were compared using the Mann Whitney U-test. Count data between groups were compared with the chi-square test. The receiver operating characteristic curve (ROC) was used to analyze the relationship between serum PCT and CSF PCT with post craniotomy intracranial infection. The sensitivity and specificity for the optimal cutoff point was calculated by the ROC curve.

## Results

### Basic data and clinical features of infected and non-infected groups

Ninety-three consecutive patients were recruited, of which 35 were confirmed with intracranial infection and 58 did not have infection. Seven patients in the infection group and 12 in the non-infection group had intracranial tumor, and 7 in the infection group and 10 in the non-infection group had spontaneous intracerebral hemorrhage. Moreover, 7 and 4 patients had craniocerebral trauma and arachnoid cyst, respectively, in the non-infection group, and 3 had craniocerebral trauma and 3 others had arachnoid cyst in the infection group. No significant difference in age, gender, APACHE II score, SOFA score, body temperature, WBC, CRP and serum PCT levels between the two groups were found (P>0.05, Table 1).

**Table 1:**
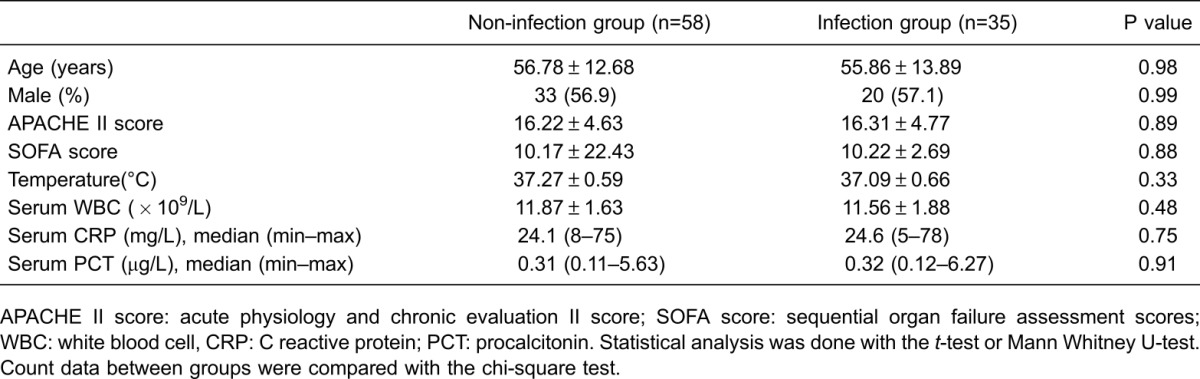
Characteristics of the 2 groups at admission

### Microbial culture results

The microbial culture results of CSF from 35 patients showed 19 positive cases (56.3%, Table 2). The infection was controlled effectively in 11 patients, while 8 still had positive bacterial culture after 3 days of treatment due to inappropriate anti-infection treatment.

**Table 2:**
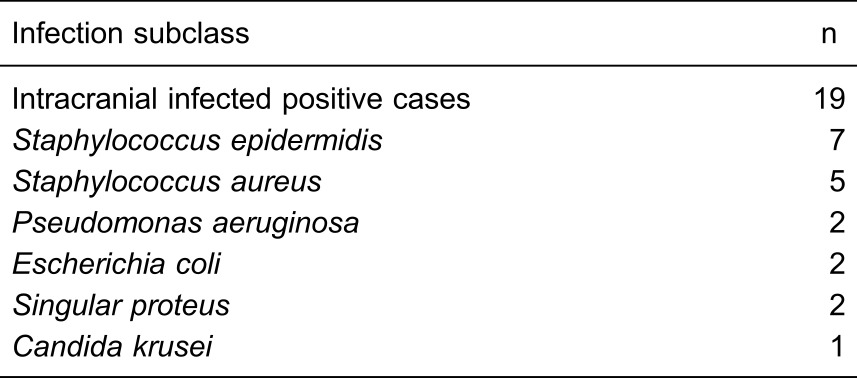
Summary of microbial culture results of 35 intracranialinfected patients

### Day 1 test results

No significant difference in SOFA scores (P>0.05) between infection group and non-infection group was observed. The body temperature, serum CRP and CSF protein levels were higher in infected group compared to non-infected group but not significantly different (P>0.05). The serum and CSF PCT, WBC, and CSF were significantly higher in infected group compared to non-infected group (P<0.05). In addition, the CSF glucose and chloride content in infected groups were significantly decreased compared to non-infected group (P<0.05, Table 3).

**Table 3:**
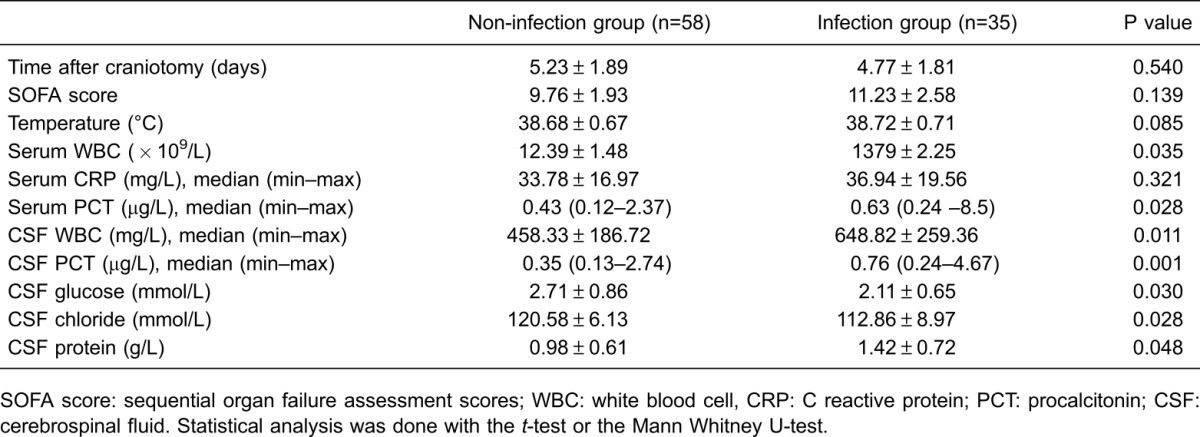
Comparison of clinical data between infection group and non-infection group at day 1

### Early diagnosis of intracranial infection by PCT and WBC

**Figure 1:**
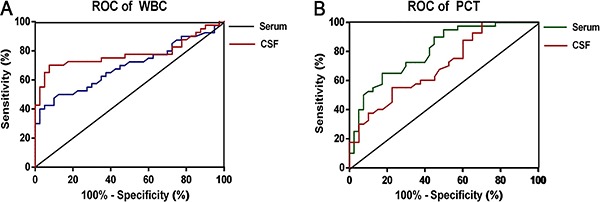
Receiver operating characteristics (ROC) curve of serum and cerebrospinal fluid (CSF) white blood cell (WBC) (*A*), and serum and CSF procalcitonin (PCT) (*B*) in diagnosis of intracranial infection after craniotomy (n=39).

ROC curve analysis showed significant differences in serum and CSF WBC and PCT (P*<*0.05, Figure 1). The areas were 0.69 (95%CI=0.58–0.81), 0.78 (95%CI=0.67–0.89), 0.70 (95%CI=0.59–0.81) and 0.80 (95%CI=0.71–0.90) for serum WBC, CSF WBC, serum PCT and CSF PCT, respectively. The diagnostic sensitivity and specificity of CSF PCT was better than other indicators. The optimized cutoff value for CSF PCT was 0.425 μg/L.

### CSF PCT level in infection-controlled and infection-uncontrolled groups

**Figure 2:**
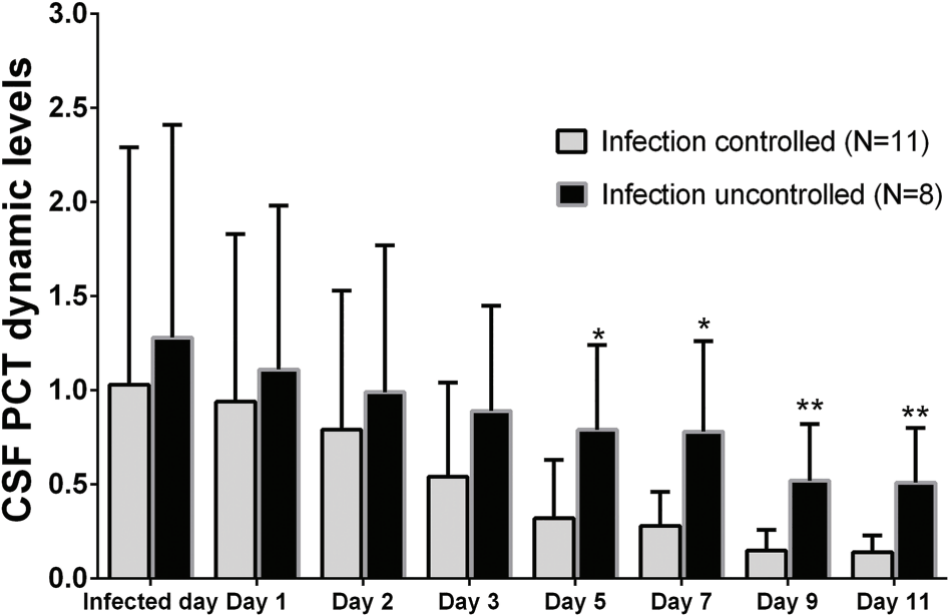
Dynamic culture of cerebrospinal fluid (CSF) procalcitonin (PCT) levels in infection-controlled group and infection-uncontrolled group from day 1 to day 11. *P<0.05, **P<0.01, compared to the infection controlled group (*t*-test).

The CSF PCT level decreased significantly in infection-controlled group after 3 days of anti-infective therapy compared with day 1 (P<0.05), and decreased subsequently in the following days (Figure 2). The CSF PCT level in infection-uncontrolled group remained high during the days of observation. A statistical difference was found for CSF PCT levels from the 5th day between infection-controlled and infection uncontrolled groups and continued until the end of the observation.

## Discussion

Intracranial infection is the most serious complication after craniotomy. Bacterial infection often causes acute meningitis, ventriculitis and brain abscess (19,20). Although craniotomy and asepsis techniques are continuously improved, intracranial infection after craniotomy still occurs sometimes (21). The risk factors are longer operative time, indwelling drainage tube, implantation of artificial materials, cerebrospinal fluid leakage, and history of diabetes, hypertension, etc. (22). A report indicated that a higher infection rate was observed in patients with operative time longer than 4 hours than in patients with shorter operative time. The infection risk in patients receiving external ventricular drainage was 9.4 times higher than in patients without it (23). Due to the disruption of the blood-brain barrier in craniotomy patients, the immune function of their central nervous system was reduced longterm, and placement of a drainage tube increased infection risk.

The use of perioperative antibiotics may reduce the incidence of postoperative infection to a certain extent, but it may also mask early clinical symptoms, leading to a difficult diagnosis. Some patients who undergo craniotomy may present symptoms that can be similar to intracranial infection as a result of the stimulation of blood and implants in the cerebrospinal fluid. Although CSF culture is the gold standard for diagnosis of intracranial infection, the time taken for culture growth and drug sensitivity tests is long and the positive rate is low, therefore diagnostic information may not be received in time for effective clinical treatment. Conventional inflammatory markers such as WBC, body temperature, and serum CRP lack specificity in the early diagnosis of intracranial infection. A diagnostic index with good sensitivity and specificity is highly essential in clinical settings to detect intracranial infection and guide clinicians to take necessary interventions, improve the prognosis of patients, and reduce the hospitalization time and economic burden. PCT has been widely studied in recent years for the early diagnosis of bacterial infection severity, treatment effect, disease prognosis and as reference for antibiotic use (24–26).

This study confirmed that serum PCT levels increase significantly after systemic bacterial infection (27), while levels are normal or only slightly elevated even in serious viral infections. Compared with serum CRP, WBC count, and percentage of neutrophils, serum PCT levels did not increase significantly in some autoimmune diseases such as rheumatoid arthritis. Other studies have indicated that high serum PCT levels are the main independent biological predictor for early diagnosis of bacterial meningitis and viral meningitis (28‗^29^). Differently from the above result, our research showed that the serum PCT levels in patients of infection group were significantly higher than in non-infection group. Compared to WBC count and serum CRP, serum PCT is a good diagnostic marker for intracranial bacterial infection. Li et al. (30) reported that combined with CSF PCT and CSF lactic acid, serum PCT could effectively identify bacterial meningitis and non-bacterial meningitis after craniotomy. In our study, ROC curve analysis showed that the diagnostic accuracy of CSF PCT is the highest among the parameters studied and it can be used as an optimal index for early diagnosis of intracranial infection after craniotomy. In the present study, the cutoff value of CSF PCT was found to be 0.425 μg/L, a little below the clinical standard of 0.5 μg/L, demonstrating its suitability for diagnosing this infection.

It has been reported that monitoring PCT levels could correctly guide antibiotic usage (31,32). In this study, we observed CSF PCT changes from day 1 to day 11. The dynamic change of CSF PCT levels demonstrated that CSF PCT can be used to evaluate antibiotic therapy after craniotomy, allowing timely dose adjustments in clinical settings according to CSF PCT levels, thus improving the patient's prognosis.

Our research is an exploratory study with many shortcomings. First, the sample size is small, making generalizations in clinical settings difficult. Second, we excluded cases with other suspected infections. Third, the enrolled patients were different from those usually encountered in the ICU, where patients’ clinical status is complex, having different infections and non-infected cases. Further research is needed to verify the accuracy of PCT in serum and CSF in the diagnosis and monitoring of intracranial infection after craniotomy, using a larger sample and systemic inflammation indexes for complex fever. In addition, as intracranial infection patients with high PCT may also have other systemic infections, it is also important to identify and exclude other causes of early infection.

In summary, compared with other inflammatory markers, serum PCT and CSF PCT levels are accurate markers for the diagnosis of intracranial infection after craniotomy. The dynamic changes of PCT levels can help adjust antibiotic treatment in clinical settings.
